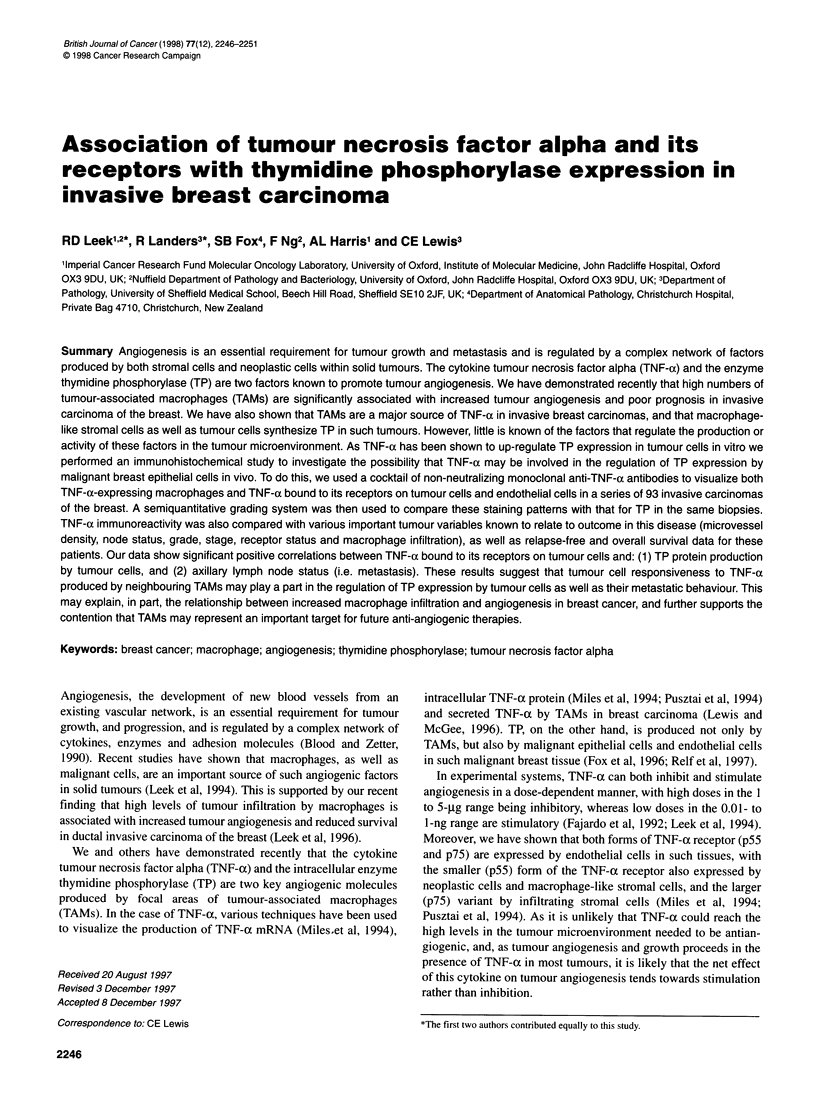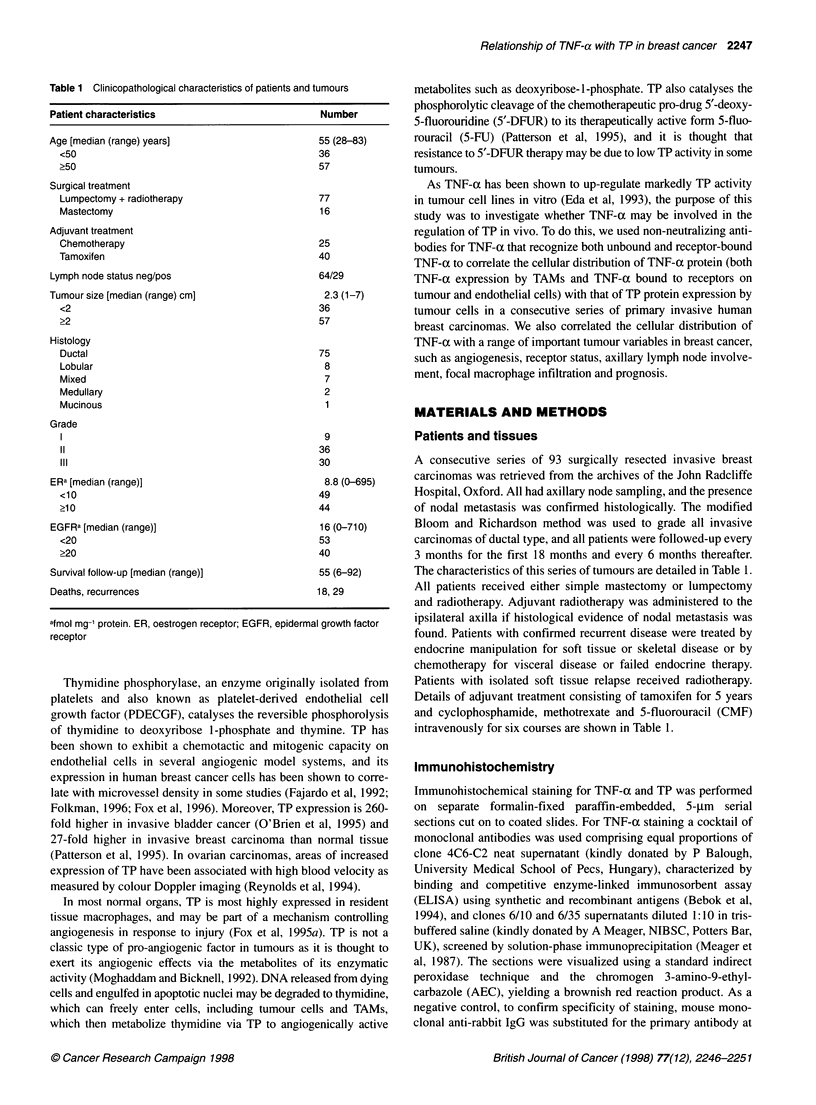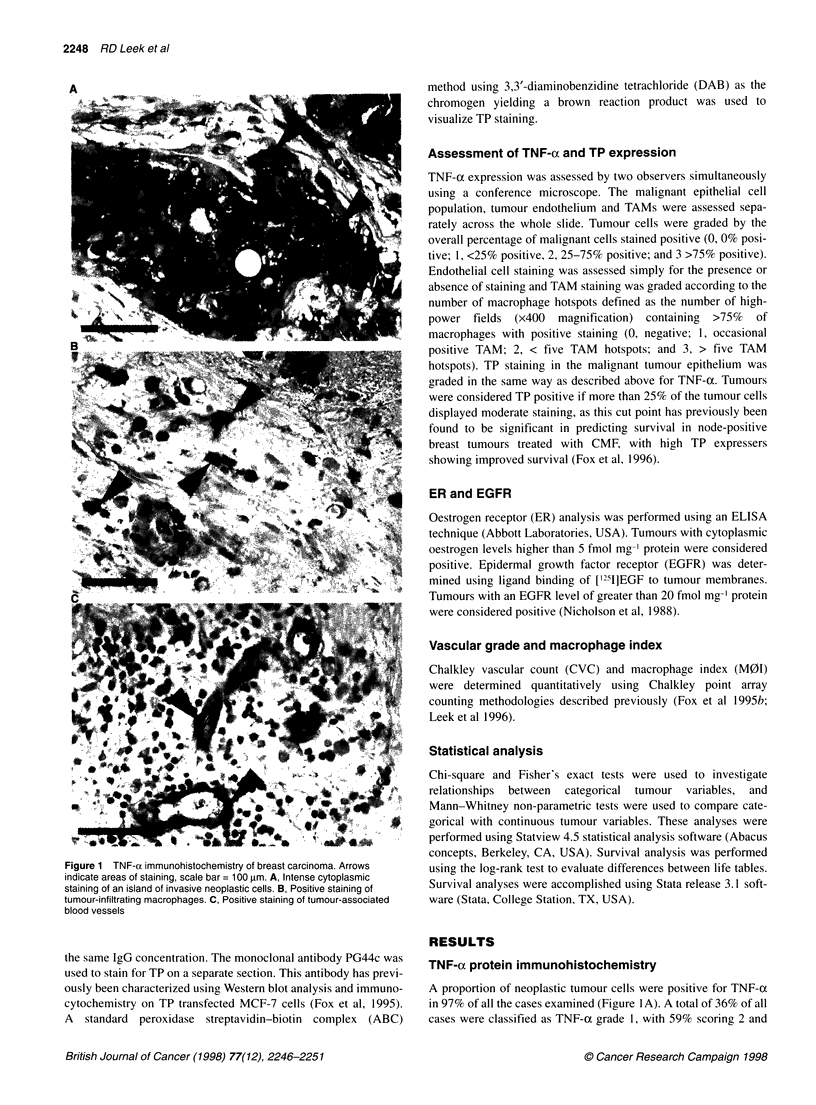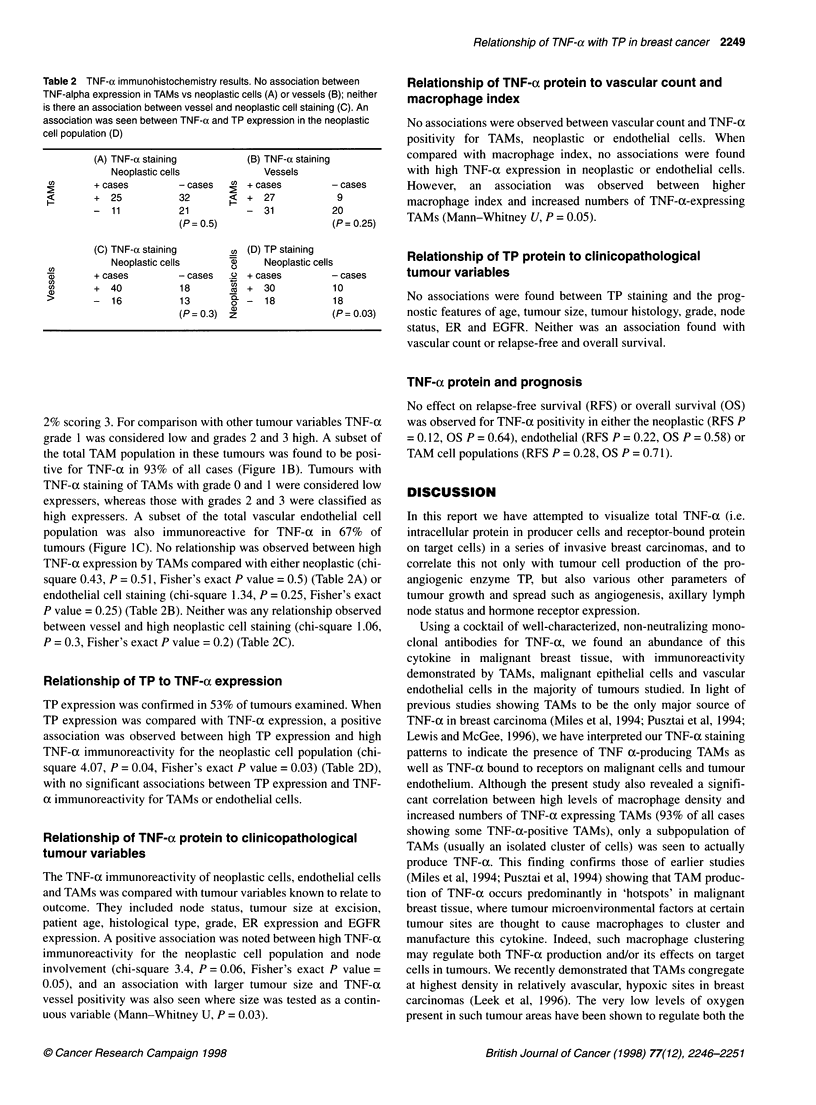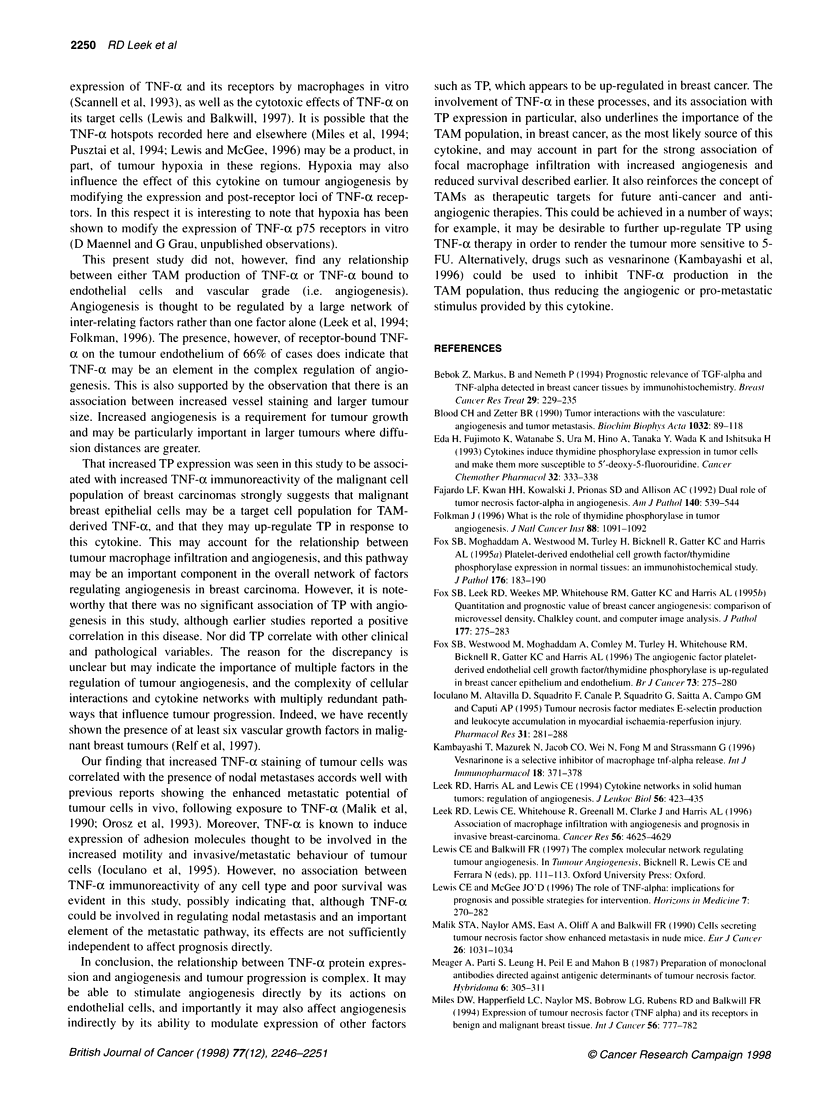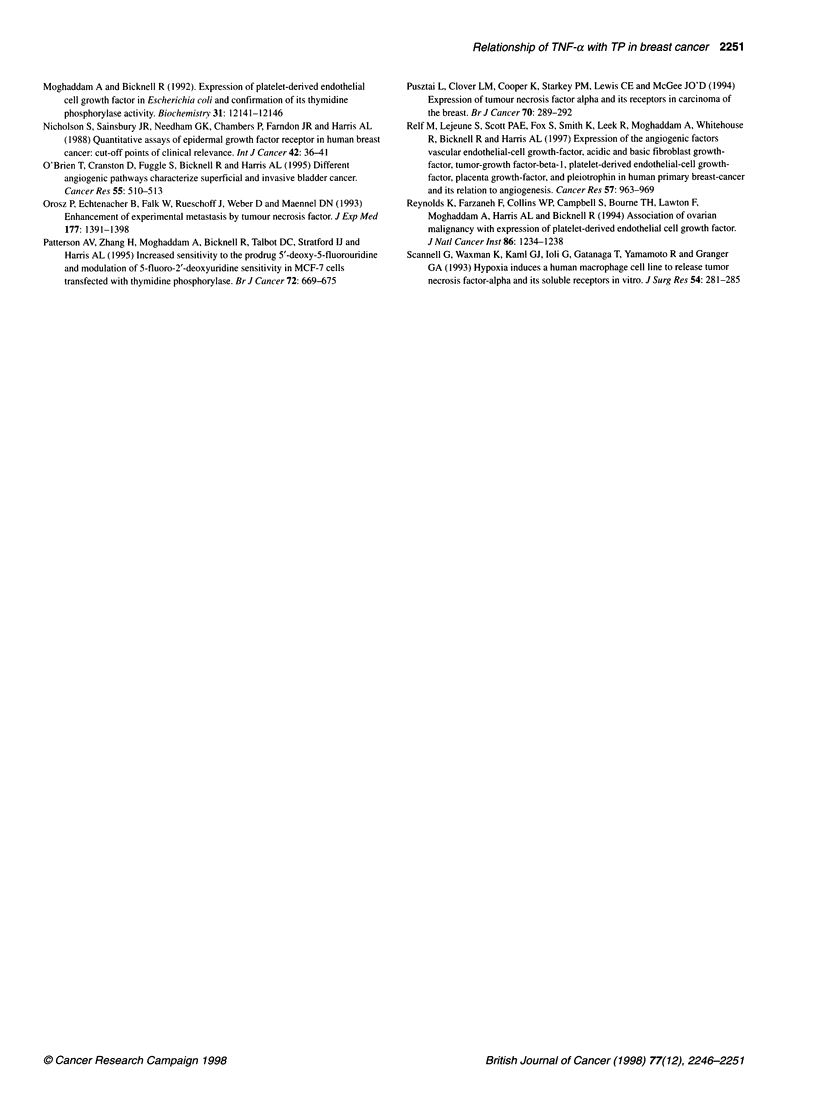# Association of tumour necrosis factor alpha and its receptors with thymidine phosphorylase expression in invasive breast carcinoma.

**DOI:** 10.1038/bjc.1998.373

**Published:** 1998-06

**Authors:** R. D. Leek, R. Landers, S. B. Fox, F. Ng, A. L. Harris, C. E. Lewis

**Affiliations:** Imperial Cancer Research Fund Molecular Oncology Laboratory, University of Oxford, Institute of Molecular Medicine, John Radcliffe Hospital, UK.

## Abstract

**Images:**


British Journal of Cancer (1998) 77(12), 2246–2251
© 1998 Cancer Research Campaign

### Association of tumour necrosis factor alpha and its receptors with thymidine phosphorylase expression in invasive breast carcinoma

RD Leek[1,2]*, R Landers[3]*, SB Fox[4], F Ng[2], AL Harris[1] and CE Lewis[3]

[1]Imperial Cancer Research Fund Molecular Oncology Laboratory, University of Oxford, Institute of Molecular Medicine, John Radcliffe Hospital, Oxford OX3 9DU, UK; [2]Nuffield Department of Pathology and Bacteriology, University of Oxford, John Radcliffe Hospital, Oxford OX3 9DU, UK; [3]Department of Pathology, University of Sheffield Medical School, Beech Hill Road, Sheffield SE10 2JF, UK; [4]Department of Anatomical Pathology, Christchurch Hospital, Private Bag 4710, Christchurch, New Zealand

**Summary** Angiogenesis is an essential requirement for tumour growth and metastasis and is regulated by a complex network of factors produced by both stromal cells and neoplastic cells within solid tumours. The cytokine tumour necrosis factor alpha (TNF-α) and the enzyme thymidine phosphorylase (TP) are two factors known to promote tumour angiogenesis. We have demonstrated recently that high numbers of tumour-associated macrophages (TAMs) are significantly associated with increased tumour angiogenesis and poor prognosis in invasive carcinoma of the breast. We have also shown that TAMs are a major source of TNF-α in invasive breast carcinomas, and that macrophage-like stromal cells as well as tumour cells synthesize TP in such tumours. However, little is known of the factors that regulate the production or activity of these factors in the tumour microenvironment. As TNF-α has been shown to up-regulate TP expression in tumour cells in vitro we performed an immunohistochemical study to investigate the possibility that TNF-α may be involved in the regulation of TP expression by malignant breast epithelial cells in vivo. To do this, we used a cocktail of non-neutralizing monoclonal anti-TNF-α antibodies to visualize both TNF-α-expressing macrophages and TNF-α bound to its receptors on tumour cells and endothelial cells in a series of 93 invasive carcinomas of the breast. A semiquantitative grading system was then used to compare these staining patterns with that for TP in the same biopsies. TNF-α immunoreactivity was also compared with various important tumour variables known to relate to outcome in this disease (microvessel density, node status, grade, stage, receptor status and macrophage infiltration), as well as relapse-free and overall survival data for these patients. Our data show significant positive correlations between TNF-α bound to its receptors on tumour cells and: (1) TP protein production by tumour cells, and (2) axillary lymph node status (i.e. metastasis). These results suggest that tumour cell responsiveness to TNF-α produced by neighbouring TAMs may play a part in the regulation of TP expression by tumour cells as well as their metastatic behaviour. This may explain, in part, the relationship between increased macrophage infiltration and angiogenesis in breast cancer, and further supports the contention that TAMs may represent an important target for future anti-angiogenic therapies.

**Keywords:** breast cancer; macrophage; angiogenesis; thymidine phosphorylase; tumour necrosis factor alpha

Angiogenesis, the development of new blood vessels from an existing vascular network, is an essential requirement for tumour growth, and progression, and is regulated by a complex network of cytokines, enzymes and adhesion molecules (Blood and Zetter, 1990). Recent studies have shown that macrophages, as well as malignant cells, are an important source of such angiogenic factors in solid tumours (Leek et al, 1994). This is supported by our recent finding that high levels of tumour infiltration by macrophages is associated with increased tumour angiogenesis and reduced survival in ductal invasive carcinoma of the breast (Leek et al, 1996).

We and others have demonstrated recently that the cytokine tumour necrosis factor alpha (TNF-α) and the intracellular enzyme thymidine phosphorylase (TP) are two key angiogenic molecules produced by focal areas of tumour-associated macrophages (TAMs). In the case of TNF-α, various techniques have been used to visualize the production of TNF-α mRNA (Miles,et al, 1994), intracellular TNF-α protein (Miles et al, 1994; Pusztai et al, 1994) and secreted TNF-α by TAMs in breast carcinoma (Lewis and McGee, 1996). TP, on the other hand, is produced not only by TAMs, but also by malignant epithelial cells and endothelial cells in such malignant breast tissue (Fox et al, 1996; Relf et al, 1997).

In experimental systems, TNF-α can both inhibit and stimulate angiogenesis in a dose-dependent manner, with high doses in the 1 to 5-μg range being inhibitory, whereas low doses in the 0.01- to 1-ng range are stimulatory (Fajardo et al, 1992; Leek et al, 1994). Moreover, we have shown that both forms of TNF-α receptor (p55 and p75) are expressed by endothelial cells in such tissues, with the smaller (p55) form of the TNF-α receptor also expressed by neoplastic cells and macrophage-like stromal cells, and the larger (p75) variant by infiltrating stromal cells (Miles et al, 1994; Pusztai et al, 1994). As it is unlikely that TNF-α could reach the high levels in the tumour microenvironment needed to be antiangiogenic, and, as tumour angiogenesis and growth proceeds in the presence of TNF-α in most tumours, it is likely that the net effect of this cytokine on tumour angiogenesis tends towards stimulation rather than inhibition.

Received 20 August 1997
Revised 3 December 1997
Accepted 8 December 1997

Correspondence to: CE Lewis

*The first two authors contributed equally to this study.

**Table 1** Clinicopathological characteristics of patients and tumours

| Patient characteristics | Number |
|---|---|
| Age [median (range) years] | 55 (28–83) |
| <50 | 36 |
| ≥50 | 57 |
| Surgical treatment | |
| Lumpectomy + radiotherapy | 77 |
| Mastectomy | 16 |
| Adjuvant treatment | |
| Chemotherapy | 25 |
| Tamoxifen | 40 |
| Lymph node status neg/pos | 64/29 |
| Tumour size [median (range) cm] | 2.3 (1–7) |
| <2 | 36 |
| ≥2 | 57 |
| Histology | |
| Ductal | 75 |
| Lobular | 8 |
| Mixed | 7 |
| Medullary | 2 |
| Mucinous | 1 |
| Grade | |
| I | 9 |
| II | 36 |
| III | 30 |
| ER[a] [median (range)] | 8.8 (0–695) |
| <10 | 49 |
| ≥10 | 44 |
| EGFR[a] [median (range)] | 16 (0–710) |
| <20 | 53 |
| ≥20 | 40 |
| Survival follow-up [median (range)] | 55 (6–92) |
| Deaths, recurrences | 18, 29 |

[a]fmol $mg^{-1}$ protein. ER, oestrogen receptor; EGFR, epidermal growth factor receptor

Thymidine phosphorylase, an enzyme originally isolated from platelets and also known as platelet-derived endothelial cell growth factor (PDECGF), catalyses the reversible phosphorolysis of thymidine to deoxyribose 1-phosphate and thymine. TP has been shown to exhibit a chemotactic and mitogenic capacity on endothelial cells in several angiogenic model systems, and its expression in human breast cancer cells has been shown to correlate with microvessel density in some studies (Fajardo et al, 1992; Folkman, 1996; Fox et al, 1996). Moreover, TP expression is 260-fold higher in invasive bladder cancer (O'Brien et al, 1995) and 27-fold higher in invasive breast carcinoma than normal tissue (Patterson et al, 1995). In ovarian carcinomas, areas of increased expression of TP have been associated with high blood velocity as measured by colour Doppler imaging (Reynolds et al, 1994).

In most normal organs, TP is most highly expressed in resident tissue macrophages, and may be part of a mechanism controlling angiogenesis in response to injury (Fox et al, 1995*a*). TP is not a classic type of pro-angiogenic factor in tumours as it is thought to exert its angiogenic effects via the metabolites of its enzymatic activity (Moghaddam and Bicknell, 1992). DNA released from dying cells and engulfed in apoptotic nuclei may be degraded to thymidine, which can freely enter cells, including tumour cells and TAMs, which then metabolize thymidine via TP to angiogenically active metabolites such as deoxyribose-1-phosphate. TP also catalyses the phosphorolytic cleavage of the chemotherapeutic pro-drug 5′-deoxy-5-fluorouridine (5′-DFUR) to its therapeutically active form 5-fluorouracil (5-FU) (Patterson et al, 1995), and it is thought that resistance to 5′-DFUR therapy may be due to low TP activity in some tumours.

As TNF-α has been shown to up-regulate markedly TP activity in tumour cell lines in vitro (Eda et al, 1993), the purpose of this study was to investigate whether TNF-α may be involved in the regulation of TP in vivo. To do this, we used non-neutralizing antibodies for TNF-α that recognize both unbound and receptor-bound TNF-α to correlate the cellular distribution of TNF-α protein (both TNF-α expression by TAMs and TNF-α bound to receptors on tumour and endothelial cells) with that of TP protein expression by tumour cells in a consecutive series of primary invasive human breast carcinomas. We also correlated the cellular distribution of TNF-α with a range of important tumour variables in breast cancer, such as angiogenesis, receptor status, axillary lymph node involvement, focal macrophage infiltration and prognosis.

### MATERIALS AND METHODS

### Patients and tissues

A consecutive series of 93 surgically resected invasive breast carcinomas was retrieved from the archives of the John Radcliffe Hospital, Oxford. All had axillary node sampling, and the presence of nodal metastasis was confirmed histologically. The modified Bloom and Richardson method was used to grade all invasive carcinomas of ductal type, and all patients were followed-up every 3 months for the first 18 months and every 6 months thereafter. The characteristics of this series of tumours are detailed in Table 1. All patients received either simple mastectomy or lumpectomy and radiotherapy. Adjuvant radiotherapy was administered to the ipsilateral axilla if histological evidence of nodal metastasis was found. Patients with confirmed recurrent disease were treated by endocrine manipulation for soft tissue or skeletal disease or by chemotherapy for visceral disease or failed endocrine therapy. Patients with isolated soft tissue relapse received radiotherapy. Details of adjuvant treatment consisting of tamoxifen for 5 years and cyclophosphamide, methotrexate and 5-fluorouracil (CMF) intravenously for six courses are shown in Table 1.

### Immunohistochemistry

Immunohistochemical staining for TNF-α and TP was performed on separate formalin-fixed paraffin-embedded, 5-μm serial sections cut on to coated slides. For TNF-α staining a cocktail of monoclonal antibodies was used comprising equal proportions of clone 4C6-C2 neat supernatant (kindly donated by P Balough, University Medical School of Pecs, Hungary), characterized by binding and competitive enzyme-linked immunosorbent assay (ELISA) using synthetic and recombinant antigens (Bebok et al, 1994), and clones 6/10 and 6/35 supernatants diluted 1:10 in tris-buffered saline (kindly donated by A Meager, NIBSC, Potters Bar, UK), screened by solution-phase immunoprecipitation (Meager et al, 1987). The sections were visualized using a standard indirect peroxidase technique and the chromogen 3-amino-9-ethyl-carbazole (AEC), yielding a brownish red reaction product. As a negative control, to confirm specificity of staining, mouse monoclonal anti-rabbit IgG was substituted for the primary antibody at

A

B

C

**Figure 1** TNF-α immunohistochemistry of breast carcinoma. Arrows indicate areas of staining, scale bar = 100 μm. **A**, Intense cytoplasmic staining of an island of invasive neoplastic cells. **B**, Positive staining of tumour-infiltrating macrophages. **C**, Positive staining of tumour-associated blood vessels the same IgG concentration. The monoclonal antibody PG44c was used to stain for TP on a separate section. This antibody has previously been characterized using Western blot analysis and immunocytochemistry on TP transfected MCF-7 cells (Fox et al, 1995). A standard peroxidase streptavidin–biotin complex (ABC) method using 3,3′-diaminobenzidine tetrachloride (DAB) as the chromogen yielding a brown reaction product was used to visualize TP staining.

### Assessment of TNF-α and TP expression

TNF-α expression was assessed by two observers simultaneously using a conference microscope. The malignant epithelial cell population, tumour endothelium and TAMs were assessed separately across the whole slide. Tumour cells were graded by the overall percentage of malignant cells stained positive (0, 0% positive; 1, <25% positive, 2, 25–75% positive; and 3 >75% positive). Endothelial cell staining was assessed simply for the presence or absence of staining and TAM staining was graded according to the number of macrophage hotspots defined as the number of high-power fields (×400 magnification) containing >75% of macrophages with positive staining (0, negative; 1, occasional positive TAM; 2, < five TAM hotspots; and 3, > five TAM hotspots). TP staining in the malignant tumour epithelium was graded in the same way as described above for TNF-α. Tumours were considered TP positive if more than 25% of the tumour cells displayed moderate staining, as this cut point has previously been found to be significant in predicting survival in node-positive breast tumours treated with CMF, with high TP expressers showing improved survival (Fox et al, 1996).

### ER and EGFR

Oestrogen receptor (ER) analysis was performed using an ELISA technique (Abbott Laboratories, USA). Tumours with cytoplasmic oestrogen levels higher than 5 fmol $mg^{-1}$ protein were considered positive. Epidermal growth factor receptor (EGFR) was determined using ligand binding of [$^{125}$I]EGF to tumour membranes. Tumours with an EGFR level of greater than 20 fmol $mg^{-1}$ protein were considered positive (Nicholson et al, 1988).

### Vascular grade and macrophage index

Chalkley vascular count (CVC) and macrophage index (MØI) were determined quantitatively using Chalkley point array counting methodologies described previously (Fox et al 1995*b*; Leek et al 1996).

### Statistical analysis

Chi-square and Fisher's exact tests were used to investigate relationships between categorical tumour variables, and Mann–Whitney non-parametric tests were used to compare categorical with continuous tumour variables. These analyses were performed using Statview 4.5 statistical analysis software (Abacus concepts, Berkeley, CA, USA). Survival analysis was performed using the log-rank test to evaluate differences between life tables. Survival analyses were accomplished using Stata release 3.1 software (Stata, College Station, TX, USA).

### RESULTS

### TNF-α protein immunohistochemistry

A proportion of neoplastic tumour cells were positive for TNF-α in 97% of all the cases examined (Figure 1A). A total of 36% of all cases were classified as TNF-α grade 1, with 59% scoring 2 and

© Cancer Research Campaign 1998

**Table 2** TNF-α immunohistochemistry results. No association between TNF-alpha expression in TAMs vs neoplastic cells (A) or vessels (B); neither is there an association between vessel and neoplastic cell staining (C). An association was seen between TNF-α and TP expression in the neoplastic cell population (D)

| | (A) TNF-α staining Neoplastic cells | | | (B) TNF-α staining Vessels | |
|---|---|---|---|---|---|
| TAMs | + cases | – cases | TAMs | + cases | – cases |
| + | 25 | 32 | + | 27 | 9 |
| – | 11 | 21 | – | 31 | 20 |
| | | ($P$ = 0.5) | | | ($P$ = 0.25) |

| | (C) TNF-α staining Neoplastic cells | | | (D) TP staining Neoplastic cells | |
|---|---|---|---|---|---|
| Vessels | + cases | – cases | Neoplastic cells | + cases | – cases |
| + | 40 | 18 | + | 30 | 10 |
| – | 16 | 13 | – | 18 | 18 |
| | | ($P$ = 0.3) | | | ($P$ = 0.03) |

2% scoring 3. For comparison with other tumour variables TNF-α grade 1 was considered low and grades 2 and 3 high. A subset of the total TAM population in these tumours was found to be positive for TNF-α in 93% of all cases (Figure 1B). Tumours with TNF-α staining of TAMs with grade 0 and 1 were considered low expressers, whereas those with grades 2 and 3 were classified as high expressers. A subset of the total vascular endothelial cell population was also immunoreactive for TNF-α in 67% of tumours (Figure 1C). No relationship was observed between high TNF-α expression by TAMs compared with either neoplastic (chi-square 0.43, $P$ = 0.51, Fisher's exact $P$ value = 0.5) (Table 2A) or endothelial cell staining (chi-square 1.34, $P$ = 0.25, Fisher's exact $P$ value = 0.25) (Table 2B). Neither was any relationship observed between vessel and high neoplastic cell staining (chi-square 1.06, $P$ = 0.3, Fisher's exact $P$ value = 0.2) (Table 2C).

### Relationship of TP to TNF-α expression

TP expression was confirmed in 53% of tumours examined. When TP expression was compared with TNF-α expression, a positive association was observed between high TP expression and high TNF-α immunoreactivity for the neoplastic cell population (chi-square 4.07, $P$ = 0.04, Fisher's exact $P$ value = 0.03) (Table 2D), with no significant associations between TP expression and TNF-α immunoreactivity for TAMs or endothelial cells.

### Relationship of TNF-α protein to clinicopathological tumour variables

The TNF-α immunoreactivity of neoplastic cells, endothelial cells and TAMs was compared with tumour variables known to relate to outcome. They included node status, tumour size at excision, patient age, histological type, grade, ER expression and EGFR expression. A positive association was noted between high TNF-α immunoreactivity for the neoplastic cell population and node involvement (chi-square 3.4, $P$ = 0.06, Fisher's exact $P$ value = 0.05), and an association with larger tumour size and TNF-α vessel positivity was also seen where size was tested as a continuous variable (Mann–Whitney U, $P$ = 0.03).

### Relationship of TNF-α protein to vascular count and macrophage index

No associations were observed between vascular count and TNF-α positivity for TAMs, neoplastic or endothelial cells. When compared with macrophage index, no associations were found with high TNF-α expression in neoplastic or endothelial cells. However, an association was observed between higher macrophage index and increased numbers of TNF-α-expressing TAMs (Mann–Whitney $U$, $P$ = 0.05).

### Relationship of TP protein to clinicopathological tumour variables

No associations were found between TP staining and the prognostic features of age, tumour size, tumour histology, grade, node status, ER and EGFR. Neither was an association found with vascular count or relapse-free and overall survival.

### TNF-α protein and prognosis

No effect on relapse-free survival (RFS) or overall survival (OS) was observed for TNF-α positivity in either the neoplastic (RFS $P$ = 0.12, OS $P$ = 0.64), endothelial (RFS $P$ = 0.22, OS $P$ = 0.58) or TAM cell populations (RFS $P$ = 0.28, OS $P$ = 0.71).

### DISCUSSION

In this report we have attempted to visualize total TNF-α (i.e. intracellular protein in producer cells and receptor-bound protein on target cells) in a series of invasive breast carcinomas, and to correlate this not only with tumour cell production of the pro-angiogenic enzyme TP, but also various other parameters of tumour growth and spread such as angiogenesis, axillary lymph node status and hormone receptor expression.

Using a cocktail of well-characterized, non-neutralizing monoclonal antibodies for TNF-α, we found an abundance of this cytokine in malignant breast tissue, with immunoreactivity demonstrated by TAMs, malignant epithelial cells and vascular endothelial cells in the majority of tumours studied. In light of previous studies showing TAMs to be the only major source of TNF-α in breast carcinoma (Miles et al, 1994; Pusztai et al, 1994; Lewis and McGee, 1996), we have interpreted our TNF-α staining patterns to indicate the presence of TNF α-producing TAMs as well as TNF-α bound to receptors on malignant cells and tumour endothelium. Although the present study also revealed a significant correlation between high levels of macrophage density and increased numbers of TNF-α expressing TAMs (93% of all cases showing some TNF-α-positive TAMs), only a subpopulation of TAMs (usually an isolated cluster of cells) was seen to actually produce TNF-α. This finding confirms those of earlier studies (Miles et al, 1994; Pusztai et al, 1994) showing that TAM production of TNF-α occurs predominantly in 'hotspots' in malignant breast tissue, where tumour microenvironmental factors at certain tumour sites are thought to cause macrophages to cluster and manufacture this cytokine. Indeed, such macrophage clustering may regulate both TNF-α production and/or its effects on target cells in tumours. We recently demonstrated that TAMs congregate at highest density in relatively avascular, hypoxic sites in breast carcinomas (Leek et al, 1996). The very low levels of oxygen present in such tumour areas have been shown to regulate both the

© Cancer Research Campaign 1998 

expression of TNF-α and its receptors by macrophages in vitro (Scannell et al, 1993), as well as the cytotoxic effects of TNF-α on its target cells (Lewis and Balkwill, 1997). It is possible that the TNF-α hotspots recorded here and elsewhere (Miles et al, 1994; Pusztai et al, 1994; Lewis and McGee, 1996) may be a product, in part, of tumour hypoxia in these regions. Hypoxia may also influence the effect of this cytokine on tumour angiogenesis by modifying the expression and post-receptor loci of TNF-α receptors. In this respect it is interesting to note that hypoxia has been shown to modify the expression of TNF-α p75 receptors in vitro (D Maennel and G Grau, unpublished observations).

This present study did not, however, find any relationship between either TAM production of TNF-α or TNF-α bound to endothelial cells and vascular grade (i.e. angiogenesis). Angiogenesis is thought to be regulated by a large network of inter-relating factors rather than one factor alone (Leek et al, 1994; Folkman, 1996). The presence, however, of receptor-bound TNF-α on the tumour endothelium of 66% of cases does indicate that TNF-α may be an element in the complex regulation of angiogenesis. This is also supported by the observation that there is an association between increased vessel staining and larger tumour size. Increased angiogenesis is a requirement for tumour growth and may be particularly important in larger tumours where diffusion distances are greater.

That increased TP expression was seen in this study to be associated with increased TNF-α immunoreactivity of the malignant cell population of breast carcinomas strongly suggests that malignant breast epithelial cells may be a target cell population for TAM-derived TNF-α, and that they may up-regulate TP in response to this cytokine. This may account for the relationship between tumour macrophage infiltration and angiogenesis, and this pathway may be an important component in the overall network of factors regulating angiogenesis in breast carcinoma. However, it is noteworthy that there was no significant association of TP with angiogenesis in this study, although earlier studies reported a positive correlation in this disease. Nor did TP correlate with other clinical and pathological variables. The reason for the discrepancy is unclear but may indicate the importance of multiple factors in the regulation of tumour angiogenesis, and the complexity of cellular interactions and cytokine networks with multiply redundant pathways that influence tumour progression. Indeed, we have recently shown the presence of at least six vascular growth factors in malignant breast tumours (Relf et al, 1997).

Our finding that increased TNF-α staining of tumour cells was correlated with the presence of nodal metastases accords well with previous reports showing the enhanced metastatic potential of tumour cells in vivo, following exposure to TNF-α (Malik et al, 1990; Orosz et al, 1993). Moreover, TNF-α is known to induce expression of adhesion molecules thought to be involved in the increased motility and invasive/metastatic behaviour of tumour cells (Ioculano et al, 1995). However, no association between TNF-α immunoreactivity of any cell type and poor survival was evident in this study, possibly indicating that, although TNF-α could be involved in regulating nodal metastasis and an important element of the metastatic pathway, its effects are not sufficiently independent to affect prognosis directly.

In conclusion, the relationship between TNF-α protein expression and angiogenesis and tumour progression is complex. It may be able to stimulate angiogenesis directly by its actions on endothelial cells, and importantly it may also affect angiogenesis indirectly by its ability to modulate expression of other factors such as TP, which appears to be up-regulated in breast cancer. The involvement of TNF-α in these processes, and its association with TP expression in particular, also underlines the importance of the TAM population, in breast cancer, as the most likely source of this cytokine, and may account in part for the strong association of focal macrophage infiltration with increased angiogenesis and reduced survival described earlier. It also reinforces the concept of TAMs as therapeutic targets for future anti-cancer and anti-angiogenic therapies. This could be achieved in a number of ways; for example, it may be desirable to further up-regulate TP using TNF-α therapy in order to render the tumour more sensitive to 5-FU. Alternatively, drugs such as vesnarinone (Kambayashi et al, 1996) could be used to inhibit TNF-α production in the TAM population, thus reducing the angiogenic or pro-metastatic stimulus provided by this cytokine.

### REFERENCES

Bebok Z, Markus, B and Nemeth P (1994) Prognostic relevance of TGF-alpha and TNF-alpha detected in breast cancer tissues by immunohistochemistry. *Breast Cancer Res Treat* **29**: 229–235

Blood CH and Zetter BR (1990) Tumor interactions with the vasculature: angiogenesis and tumor metastasis. *Biochim Biophys Acta* **1032**: 89–118

Eda H, Fujimoto K, Watanabe S, Ura M, Hino A, Tanaka Y, Wada K and Ishitsuka H (1993) Cytokines induce thymidine phosphorylase expression in tumor cells and make them more susceptible to 5′-deoxy-5-fluorouridine. *Cancer Chemother Pharmacol* **32**: 333–338

Fajardo LF, Kwan HH, Kowalski J, Prionas SD and Allison AC (1992) Dual role of tumor necrosis factor-alpha in angiogenesis. *Am J Pathol* **140**: 539–544

Folkman J (1996) What is the role of thymidine phosphorylase in tumor angiogenesis. *J Natl Cancer Inst* **88**: 1091–1092

Fox SB, Moghaddam A, Westwood M, Turley H, Bicknell R, Gatter KC and Harris AL (1995*a*) Platelet-derived endothelial cell growth factor/thymidine phosphorylase expression in normal tissues: an immunohistochemical study. *J Pathol* **176**: 183–190

Fox SB, Leek RD, Weekes MP, Whitehouse RM, Gatter KC and Harris AL (1995*b*) Quantitation and prognostic value of breast cancer angiogenesis: comparison of microvessel density, Chalkley count, and computer image analysis. *J Pathol* **177**: 275–283

Fox SB, Westwood M, Moghaddam A, Comley M, Turley H, Whitehouse RM, Bicknell R, Gatter KC and Harris AL (1996) The angiogenic factor platelet-derived endothelial cell growth factor/thymidine phosphorylase is up-regulated in breast cancer epithelium and endothelium. *Br J Cancer* **73**: 275–280

Ioculano M, Altavilla D, Squadrito F, Canale P, Squadrito G, Saitta A, Campo GM and Caputi AP (1995) Tumour necrosis factor mediates E-selectin production and leukocyte accumulation in myocardial ischaemia-reperfusion injury. *Pharmacol Res* **31**: 281–288

Kambayashi T, Mazurek N, Jacob CO, Wei N, Fong M and Strassmann G (1996) Vesnarinone is a selective inhibitor of macrophage tnf-alpha release. *Int J Immunopharmacol* **18**: 371–378

Leek RD, Harris AL and Lewis CE (1994) Cytokine networks in solid human tumors: regulation of angiogenesis. *J Leukoc Biol* **56**: 423–435

Leek RD, Lewis CE, Whitehouse R, Greenall M, Clarke J and Harris AL (1996) Association of macrophage infiltration with angiogenesis and prognosis in invasive breast-carcinoma. *Cancer Res* **56**: 4625–4629

Lewis CE and Balkwill FR (1997) The complex molecular network regulating tumour angiogenesis. In *Tumour Angiogenesis*, Bicknell R, Lewis CE and Ferrara N (eds), pp. 111–113. Oxford University Press: Oxford.

Lewis CE and McGee JO'D (1996) The role of TNF-alpha: implications for prognosis and possible strategies for intervention. *Horizons in Medicine* **7**: 270–282

Malik STA, Naylor AMS, East A, Oliff A and Balkwill FR (1990) Cells secreting tumour necrosis factor show enhanced metastasis in nude mice. *Eur J Cancer* **26**: 1031–1034

Meager A, Parti S, Leung H, Peil E and Mahon B (1987) Preparation of monoclonal antibodies directed against antigenic determinants of tumour necrosis factor. *Hybridoma* **6**: 305–311

Miles DW, Happerfield LC, Naylor MS, Bobrow LG, Rubens RD and Balkwill FR (1994) Expression of tumour necrosis factor (TNF alpha) and its receptors in benign and malignant breast tissue. *Int J Cancer* **56**: 777–782

© Cancer Research Campaign 1998

Moghaddam A and Bicknell R (1992). Expression of platelet-derived endothelial cell growth factor in *Escherichia coli* and confirmation of its thymidine phosphorylase activity. *Biochemistry* **31**: 12141–12146

Nicholson S, Sainsbury JR, Needham GK, Chambers P, Farndon JR and Harris AL (1988) Quantitative assays of epidermal growth factor receptor in human breast cancer: cut-off points of clinical relevance. *Int J Cancer* **42**: 36–41

O'Brien T, Cranston D, Fuggle S, Bicknell R and Harris AL (1995) Different angiogenic pathways characterize superficial and invasive bladder cancer. *Cancer Res* **55**: 510–513

Orosz P, Echtenacher B, Falk W, Rueschoff J, Weber D and Maennel DN (1993) Enhancement of experimental metastasis by tumour necrosis factor. *J Exp Med* **177**: 1391–1398

Patterson AV, Zhang H, Moghaddam A, Bicknell R, Talbot DC, Stratford IJ and Harris AL (1995) Increased sensitivity to the prodrug 5′-deoxy-5-fluorouridine and modulation of 5-fluoro-2′-deoxyuridine sensitivity in MCF-7 cells transfected with thymidine phosphorylase. *Br J Cancer* **72**: 669–675

Pusztai L, Clover LM, Cooper K, Starkey PM, Lewis CE and McGee JO'D (1994) Expression of tumour necrosis factor alpha and its receptors in carcinoma of the breast. *Br J Cancer* **70**: 289–292

Relf M, Lejeune S, Scott PAE, Fox S, Smith K, Leek R, Moghaddam A, Whitehouse R, Bicknell R and Harris AL (1997) Expression of the angiogenic factors vascular endothelial-cell growth-factor, acidic and basic fibroblast growth-factor, tumor-growth factor-beta-1, platelet-derived endothelial-cell growth-factor, placenta growth-factor, and pleiotrophin in human primary breast-cancer and its relation to angiogenesis. *Cancer Res* **57**: 963–969

Reynolds K, Farzaneh F, Collins WP, Campbell S, Bourne TH, Lawton F, Moghaddam A, Harris AL and Bicknell R (1994) Association of ovarian malignancy with expression of platelet-derived endothelial cell growth factor. *J Natl Cancer Inst* **86**: 1234–1238

Scannell G, Waxman K, Kaml GJ, Ioli G, Gatanaga T, Yamamoto R and Granger GA (1993) Hypoxia induces a human macrophage cell line to release tumor necrosis factor-alpha and its soluble receptors in vitro. *J Surg Res* **54**: 281–285

© Cancer Research Campaign 1998